# Halotolerant and plant growth-promoting endophytic fungus *Aspergillus terreus* CR7 alleviates salt stress and exhibits genoprotective effect in *Vigna radiata*

**DOI:** 10.3389/fmicb.2024.1336533

**Published:** 2024-02-09

**Authors:** Pooja Chauhan, Mandeep Singh, Avinash Sharma, Mangaljeet Singh, Pooja Chadha, Amarjeet Kaur

**Affiliations:** ^1^Department of Microbiology, Guru Nanak Dev University, Amritsar, India; ^2^Department of Zoology, Guru Nanak Dev University, Amritsar, India; ^3^Department of Biotechnology, Guru Nanak Dev University, Amritsar, India

**Keywords:** endophytes, salt stress, growth promotion, ACC deaminase, *Aspergillus terreus*, *Vigna radiata*

## Abstract

Soil salinity is one of the major environmental stresses that results in reduction of cultivable land and decreased productivity. In the present study, halotolerant and plant growth-promoting endophytic fungi were isolated from *Catharanthus roseus*, and their effect in mitigating salt stress in *Vigna radiata* was evaluated. An isolate CR7, identified to be *Aspergillus terreus*, showing plant growth promotion activities, viz. IAA production (23.43 ± 0.79 μg/ml), phosphate solubilization (133.63 ± 6.40 μg/ml), ACC deaminase activity (86.36 ± 2.70 μmol α-ketobutyrate/h/mg protein) etc. and ability to grow at 15% NaCl was selected for further *in vivo* studies. Colonization of CR7 was carried out in *V. radiata* which was subjected to different concentrations of salt (150, 200, and 250 mM NaCl). Under salt stress, *A. terreus* CR7 inoculated plants showed substantially improved root and shoot length, biomass, chlorophyll content, relative water content, phenolics, protein content, and DPPH scavenging activity. Endogenous IAA level was enhanced by 5.28-fold in treated plants at maximum salt stress. Inoculation of *A. terreus* CR7 affected oxidative stress parameters, exhibiting an increase in catalase and superoxide dismutase and reduction in proline, electrolyte leakage, and malondialdehyde content. Fluorescent microscopic analysis of roots revealed improved cell viability and decreased levels of glutathione and hydrogen peroxide under salt stress in treated plants. The isolate *A. terreus* CR7 also protected against DNA damage induced by salt stress which was evaluated using comet assay. A decrease in DNA tail length, tail moment, and olive tail moment to the extent of 19.87%, 19.76%, and 24.81%, respectively, was observed in *A. terreus* CR7-colonized plants under salt stress. It can be concluded that *A. terreus* CR7 can be exploited for alleviating the impact of salt stress in crop plants.

## 1 Introduction

Soil salinity is a major significant global problem that leads to reduced agricultural production and threats crop yield. Moreover, salt stress in agricultural land is predicted to elevate further as a result of climate change, industrial waste released into soil, excessive use of agro-chemicals, widespread introduction of low-quality water, and intensive farming (Machado and Serralheiro, 2017; Hussain H. A. et al., 2019; Ullah et al., 2021). Salt stress constrains crop yield by activating an osmotic and ionic imbalance inside plant cells. Plant growth triggered by salt stress disturbs morphological, physiological, and molecular attributes, along with increased oxidative stress that subsequently retards plant productivity (Bibi et al., 2019; Hussain S. et al., 2019). Elevated levels of Na^+^ in plant cells disrupt K^+^/Na^+^ balance, hamper uptake of water, and trigger the formation of reactive oxygen species (ROS) affecting metabolism of plants (Parihar et al., 2015; Gul Jan et al., 2019). High saline environment for plants has also shown to increase levels of 1- aminocyclopropane-1-carboxylic acid (ACC), the precursor of ethylene which promotes epinasty (Ali et al., 2014; Müller and Munné-Bosch, 2015). Although several conventional techniques can be employed to reduce soil salinity, such as better drainage management, leaching, surface flushing, irrigation of freshwater, and chemical treatments, yet reclamation of salt-affected land is financially expensive along with scarce availability of acceptable quality of irrigation water (Qadir et al., 2008). Thus, agricultural production in these saline environments requires development of crops with high salt tolerance. Conventional breeding and genetic alteration techniques to develop salinity-resistant plant species have met with limited success, necessitating the development of novel innovative approaches. In the last few decades, scientists across the world have been working to find a sustainable solution to this which is cost-effective, quick to implement, and can mitigate salt-related problems in crops to enhance agricultural productivity and sustainability under various environmental stresses (Mbarki et al., 2018; Moghimi et al., 2018). Plant–microbe interactions, especially the use of endophytes, are an effective approach to enhance agricultural output in both normal and stress conditions (White et al., 2019; Gorai et al., 2020; Mengistu, 2020; Anand et al., 2023). Endophytes are defined as “the microorganisms which spend a whole or part of their life cycle colonizing inter- or intracellularly, healthy living tissues of the host without causing any symptoms” (Petrini, 1991). Endophytic fungi supply secondary metabolites and raise tolerance against abiotic stresses after boosting the defense mechanism (Galeano et al., 2021; Gupta et al., 2021). Endophytic fungi have the capability to produce an array of bioactive components, promote plant growth, enhance accessibility of nutrients, and improve plant fitness by increasing water-use efficiency. Endophytic fungi produce various metabolites such as polyphenols, flavonoids, and phytohormones (auxin, gibberellins, and cytokinin, etc.) that greatly influence metabolism, reproduction, and overall growth of plant in various stresses (Baron and Rigobelo, 2022; Jan et al., 2022). Endophytic fungi also exhibit ACC deaminase activity which subsequently limits ethylene production (Singh et al., 2015). Colonization of plants with endophytic fungi has been reported to mitigate salt effects on plant development by various mechanisms, *viz*. increasing levels of protective metabolites and osmoprotectants, producing antioxidants to prevent ROS damage, reducing root respiration induced by salt, and improving phytohormone profile (Waqas et al., 2012; Zhang F. et al., 2019; Gupta et al., 2021). The use of endophytic fungi can prove to be a sustainable and cost-effective approach to enhance the salt tolerance of commercially important crops. Therefore, the current study aimed at isolation of endophytic fungi from *Catharanthus roseus*, collected from fields with high salinity stress and screening them for plant growth promotion and halotolerance abilities. The culture exhibiting good plant growth promotion properties and halotolerance was subjected to *in vivo* studies for its ability to mitigate salt stress in *Vigna radiata*. *Vigna radiata* is one of the most important and highly cultivated summer crops and is considered valuable due to its medicinal and nutritional properties. Its biochemical characteristics such as germination, growth rate, biomass, protein, and carbohydrate are affected by salt stress (Sehrawat et al., 2019). Effect of colonization of selected culture in *V. radiata* plants was determined on various physiological and biochemical parameters under salt stress. Salinity stress can also result in DNA damage in plant tissue (Alotaibi, 2021; Darwish et al., 2023). High salinity-induced oxidative stress is characterized as generation of ROS and imbalance of antioxidants molecules (AbdElgawad et al., 2016). One of the major outcomes of persistent oxidative stress is DNA damage (Barzilai and Yamamoto, 2004). Antioxidants are the molecules which prevent the formation of ROS by scavenging and converting them to less reactive molecules (Santos-Sánchez et al., 2019). Endophytic fungi have the potential to produce antioxidants that prevent ROS generation and protect DNA damage (Kaur et al., 2020). Genoproptective effect of selected culture in plants exposed to salt stress was also evaluated using comet assay.

## 2 Materials and methodology

### 2.1 Isolation of endophytic fungi

Isolation of endophytic fungi was carried out from healthy asymptomatic plant parts of *C. roseus*. To remove solid and attached debris from plant parts, running tap water was used. Surface sterilization was attained by immersing plant parts in 70% ethanol (1–2 min), rinsing with sterile distilled water followed by immersion in 4% sodium hypochlorite (2–3 min), and then washing again with sterile distilled water. Surface-sterilized plant parts were cut with sterilized blade into small fragments (2–5 mm size) and placed on water agar medium supplemented with chloramphenicol (100 mg/L) and incubated at 30°C for 3–5 days to several weeks. The fungal hyphae that emerged from the sterilized plant parts were picked, purified, and preserved on Potato Dextrose Agar (PDA) slants (Singh and Kaur, 2016).

### 2.2 Screening of isolated endophytes for plant growth promotion traits

#### 2.2.1 Indole-3-acetic acid production

To estimate production of IAA, fungal cultures were inoculated in Czapek Dox broth supplemented with 2 mg/ml L-tryptophan and incubated at 30°C. Un-inoculated medium containing L-tryptophan was used as control. After incubation, IAA synthesis by fungal endophytes was determined by mixing 1 ml of culture supernatant with 2 ml of Salkowski reagent. After incubating for 25 min in dark at room temperature, absorbance was measured at 530 nm. For measuring IAA production, a standard curve of IAA was prepared (Gordon and Weber, 1951).

#### 2.2.2 Phosphate solubilization

Pikovskaya's (1948) agar medium was used to determine phosphate solubilization ability. The fungal cultures were inoculated and kept at 30°C until growth appeared. After incubation, the development of a halo zone or clearing surrounding the colony indicated the potential to solubilize phosphate. The phosphate solubilization index (PSI) was determined using the Equation 1:


(1) $$\text{PSI}=\frac{\text{Colony diameter }+\text{Halozone diameter}}{\text{Colony diameter}}$$

#### 2.2.3 Siderophore production

The Chrome Azurol S (CAS) method, developed by Schwyn and Neilands (1987), was used for siderophore analysis. The isolates were inoculated in CAS agar medium and incubated for 4 days at 30°C. Pink color zone formed around the colonies indicated the production of siderophores.

#### 2.2.4 Hydrogen cyanide production

For determining hydrogen cyanide production, fungal isolates were inoculated in PDA medium supplemented with 0.4% (w/v) glycine. A sterilized Whatman filter paper soaked in alkaline solution (0.5% picric acid and 2% sodium carbonate) was placed underneath the petri plates lids inoculated with fungal isolates. The plates were sealed with parafilm and incubated at 30°C for 7 days. Color change from yellow to brown indicated a positive result (Bakker and Schippers, 1987).

#### 2.2.5 Ammonium production

Ammonia production was estimated by using Nessler's reagent in peptone liquid medium. The appearance of brown color indicated the capacity for ammonia production (Cappucino and Sherman, 1992).

#### 2.2.6 Salt stress tolerance

The fungal isolates were evaluated for their salt tolerance ability by growing on PDA medium supplemented with different concentrations of NaCl (0%, 5%, 10%, and 15%) for 10 days at 30°C.

### 2.3 Molecular identification of CR7 fungal isolate

The isolate CR7 showing good plant growth promotion activity and halotolerance was selected for further studies and identified on morphological and molecular basis. Slide culturing method was used for morphological characterization and identification using standard taxonomic key characters. Molecular characterization was performed by amplification of ITS1-5.8S rDNA-ITS2 region using universal primers. The purified ITS region was sequenced by Gene Matrix LLP, Pune, India. The phylogenetic tree was constructed in MEGA10 software using neighbor-joining method, and the evolutionary distances were computed using the maximum composite likelihood method. The culture has been submitted to National Center for Microbial Resource (NCMR) at National Center for Cell Science (NCCS), Pune, Maharashtra, India.

### 2.4 Estimation of IAA production by isolate CR7

The effect of different concentrations of tryptophan and incubation time on IAA production by isolate CR7 was determined. The culture was grown in Czapek Dox broth supplemented with different concentrations of tryptophan (2–10 mg/ml) and estimated for IAA production at regular interval of 24 h up to 7 days.

### 2.5 Estimation of phosphate solubilization by isolate CR7

The estimation of phosphate solubilization was determined by inoculating CR7 in Pikovskaya's broth containing tri-calcium phosphate and incubated at 30°C. Solubilized phosphate in culture supernatant was assessed periodically every 7 days for 35 days by method of Murphy and Riley (1962). pH of cell free supernatant was also measured.

### 2.6 Aminocyclopropane-1-carboxylate deaminase production by isolate CR7

Selected fungal culture CR7 was inoculated in Dwarkin and Foster medium supplemented with ACC as a nitrogen source. Ammonium sulfate containing medium was used as control, and plates were incubated at 30°C for 5 days. The quantitative measurement of aminocyclopropane-1-carboxylate deaminase (ACCD) synthesis was determined by inoculating the fungal culture CR7 in 10 ml synthetic medium (composition per liter: glucose, 15 g; MgSO_4_.7H_2_O, 0.2 g; K_2_HPO_4_, 0.6 g; KCl, 0.15 g; NH_4_NO_3_, 1 g; 1 ml of trace solution containing per liter: FeSO_4_.7H_2_O, 0.005 g; MnSO_4_.H_2_O, 0.006 g; ZnSO_4_.H_2_O, 0.004 g; CoCl_2_, 0.002 g) (Yedidia et al., 1999) and incubated for 2 days at 30°C. Following incubation, the culture broth was centrifuged (6,000 rpm for 5 min). The supernatant was discarded, and the pellet was mixed with 5 ml of sterile synthetic medium containing 3mM concentration of ACC but lacking ammonium nitrate (NH_4_NO_3_) and incubated for 2 days at 30°C. After incubation, the culture broth was again subjected to centrifugation (6,000 rpm for 5 min). The supernatant was discarded, and the pellet was suspended in 2.5 ml of Tris-Cl buffer (0.1 M, pH 8.5). ACCD activity was measured as per the method given by Nascimento et al. (2019). The concentration of alpha-ketobutyrate released was determined by using a standard curve of pure alpha-ketobutyrate. Enzymatic activity was expressed as μmol alpha-ketobutyrate/h/mg protein. Protein was estimated using Bradford method (Bradford, 1976). For further verification, Fourier-transform infrared spectroscopy (FTIR) was used to confirm the release of alpha-ketobutyrate formed by deamination reaction involving the sole nitrogen source ACC.

### 2.7 Colonization of fungal isolate CR7 in *V. radiata*

Uniform and healthy seeds of *V. radiata* were selected and surface-sterilized with 70% ethanol, rinsed with deionized distilled water, and allowed to germinate at 25°C ± 2 on Whatman filter paper in petri dishes. After 2 days, germinated seeds were immersed in fungal spore suspension (1 × 10^8^ conidia ml^−1^) for 8 h. After treatment, seeds were air-dried on a sterile paper for 20 min and transferred to autoclaved soil. Water-treated seeds containing 0.05% Triton X-100 served as a control.

#### 2.7.1 Assessment of colonization

To determine the colonization of endophytic fungal isolates in *V. radiata*, plants were uprooted after 7 days and then washed gently with water. Isolation was carried out on potato dextrose agar medium supplemented with 15% NaCl which served as a marker for the selection of inoculated halotolerant endophytic fungus, using standard protocol (Singh and Kaur, 2016). The obtained isolates were subjected to morphological observation and compared with mother culture. Percentage of colonization in plant parts was calculated by using Equation 2:


(2) $$\text{Colonization}(\%)=\frac{\text{PF}}{\text{TP}}\times 100$$

where, PF, number of plant pieces with fungal growth; TP, total number of plant parts plated out.

### 2.8 Pot experiments

Plant growth promotion and salt stress mitigation in successfully colonized plants were determined by subjecting them to salt stress. The experiment was carried out in triplicate, and each replicate comprised of three pots with four seedlings (total 4 × 3 × 3 = 36 plants per treatment). Salinity stress of 50 ml of water containing 150, 200, and 250 mM NaCl was applied twice a week in all sets of pots, except the controls.

Experimental setup

1. Control plants (no salt stress + no fungal inoculation).
2. Plants treated with different concentrations of NaCl (150, 200, 250 mM + no fungal inoculation).
3. Fungal inoculated plants (no salt stress).
4. Fungal inoculated plants treated with different concentrations of NaCl (150, 200, 250 mM + fungal inoculation).

#### 2.8.1 Determination of growth parameters after salt stress

The plants were harvested after 20-days experiment, and the roots were washed with distilled water to remove any residual soil and debris. The root, shoot length, and fresh dry weight of plants grown under different treatments were determined by using standard measuring scale.

#### 2.8.2 Estimation of photosynthetic pigments

Fresh leaf tissue was ground in 80% acetone in 1:4 ratio and centrifuged at 4°C for 20 min at 10,000 rpm. The supernatant was collected and absorbance was recorded at wavelengths of 645 and 663 nm for chlorophyll estimation, whereas absorbance was recorded at 480 and 510 nm for calculating carotenoid content (Arnon, 1949). Photosynthetic pigments were calculated by using Equation 3:


(3) $$\begin{array}{l} \text{Chlorophyll a}=12.7(\text{A663})-2.29(\text{A645})\\ \text{Chlorophyll b}=22.9(\text{A645})-4.68(\text{A663})\\ \text{Total Chlorophyll}=20.2(\text{A645})+8.02(\text{A663})\\ \text{Carotenoid content}=0.304(\text{A480})-0.0596(\text{A510}) \end{array}$$

#### 2.8.3 Determination of RWC and REC

Relative water content (RWC) of leaf was based on the weight of fresh biomass (FW), dry biomass (DW), and moist biomass (TW) before and after leaf samples were dried. Leaf relative water content was determined using the Equation 4 (Balestri et al., 2014),


(4) $$\text{RWC}=\frac{\text{FW}-\text{DW}}{\text{TW}-\text{DW}}$$

Relative electrical conductivity (REC) of leaf sample was determined using the methodology given by Lutts et al. (1996). One cm^2^ pieces of leaf sample was immersed in 10 ml distilled deionized water in test tubes, and electric conductivity was measured at room temperature (EC1). Then, the tubes were incubated at 40°C for 25 min, and electrical conductivity was measured (EC2). After that, tubes were incubated at 100°C for 25 min and electrical conductivity was again measured (EC3). Final electrical conductivity was calculated using Equation 5:


(5) $$\text{EL}(\%)=\frac{\text{EC2}-\text{EC1}}{\text{EC3}}\times 100$$

#### 2.8.4 Estimation of proline content

The proline content was evaluated using the methodology given by Bates et al. (1973). The plant sample (0.5 g) was homogenized in 10 ml of sulfosalicylic acid (3%) and centrifuged for 15 min at 5,000 rpm. Equal volume of glacial acetic acid and acid ninhydrin was added to the supernatant, and the mixture was boiled at 100°C for 1 h in a water bath. After that, sample mixture was immediately placed in ice followed by the addition of toluene after 2–3 min. Absorbance of the upper layer was measured at 520 nm. The proline content was expressed in milligram per gram of fresh tissue using a stock solution of L-proline at a concentration of 0.1 mg/ml.

#### 2.8.5 Malondialdehyde content

Malondialdehyde (MDA) content was determined using the methodology given by Heath and Packer (1968). 0.5 g of plant tissue was homogenized in 0.1% trichloroacetic acid (TCA) and centrifuged at 4°C. One hundred microliter of supernatant was mixed with 600 μl of 20% TCA containing 0.5% thiobarbituric acid. The reaction mixture was heated at 95°C for 30 min in water bath and immediately cooled in ice. The absorbance of clear solution was taken at 532 and 600 nm. MDA content was expressed in μmol/g of plant tissue.

#### 2.8.6 Quantification of endogenous IAA levels

IAA was quantified by grinding 0.5 g of plant sample in 10 ml distilled water and centrifuged at 1,000 rpm for 15 min. Reaction mixture containing 1 ml clear supernatant and 2 ml Salkowski reagent was incubated in the dark for 25 min, and absorbance was measured at 530 nm through a UV–VIS spectrophotometer.

#### 2.8.7 Total phenolic content and DPPH activity

To determine the total phenolic content and DPPH activity, 0.5 g leaf sample was homogenized in 80% methanol and then centrifuged at 10,000 rpm for 20 min. 100 μl of culture supernatant was mixed with 2.9 ml distilled water, 500 μl of FC reagent: water (1:1), and 2 ml of 20% sodium carbonate. The mixture was then shaken vigorously, and absorbance was measured at a wavelength of 760 nm (Malik and Singh, 1980).

DPPH free radical scavenging activity was determined by following standard protocol described by Blois (1958). One hundred microliter DPPH solution (0.2 mM in methanol) was mixed with 100 μl extract (dissolved in methanol). In control, 100 μl methanol was added in 100 μl DPPH. Reaction mixture was incubated at 37°C for 30 min, and decrease in absorbance was recorded at 517 nm. Radical scavenging activity was calculated by Equation 6:


(6) $$\text{Radical scavenging activity}(\%)=\frac{\text{Ac}-\text{As}}{\text{Ac}}\times 100$$

#### 2.8.8 Estimation of protein content and antioxidant enzymes

For quantification of total protein and antioxidant enzymes, leaf samples were homogenized in 0.1 M potassium phosphate buffer (pH 6.8) using chilled mortar and pestle. The homogenate mixture was centrifuged at 1000 rpm for 15 min, and supernatant was used for estimation of protein and antioxidant enzymes. Total protein content was estimated using protocol (Bradford, 1976). Catalase and superoxide dismutase activities were calculated by using the protocols as given by Aebi (1974) and Kono (1978), respectively.

### 2.9 Fluorescent microscopic studies

The viability of the cells was determined by dipping the root tips of *V. radiata* in 50 μM solution of propidium iodide, for 15 min at room temperature. The dyed root tips were washed with double distilled water to remove the excess PI, mounted on a slide, and examined under Olympus BX43 (Tokyo, Japan) fluorescent microscope at 543 and 617 nm for excitation and emission wavelength, respectively.

For localization of hydrogen peroxide, root tips were immersed in 50 μM dichlorofluorescein-diacetate (DCF-DA) for 1 h in the dark and washed with deionized water. After that, samples were put on glass slides and examined under fluorescent microscope at wavelengths of 485 and 535 nm for excitation and emission, respectively.

To tag glutathione, root tips were dipped in 25 μM monochlorobimane (MCB) solution, left in the dark for 1 h and remaining stain was removed with deionized water. After that, samples were put on glass slides and examined under fluorescent microscope with excitation and emission at wavelengths of 351–364 and 477 nm, respectively.

### 2.10 Genoprotective effect of *A. terreus* CR7 in roots of *V. radiata*

Comet assay was used to assess the genoprotective effect of *A. terreus* CR7 expose to salt stress. The modified method of Tice et al. (2000) was applied for the determination of DNA damage induced by salt stress. The un-inoculated and *A. terreus* CR7 inoculated plants exposed to highest salt stress (250 mM) and non- salt stress groups were used for root samples. The nuclei were isolated in 600 μl ice-cold nuclear isolation buffer (pH 7.5, 4 mM MgCl_2_-6H_2_O, 0.5% w/v Triton X-100, 200 mM Tris) in a petri plate over ice. After centrifugation, 50 μl nuclear suspension and 100 μl of 0.5% low melting point agarose (LMPA) in phosphate buffer saline (pH 7.4) were pipetted on pre-coated slides (1% normal melting point agarose kept at 37°C overnight). The gel was allowed to solidify for 5–6 min on ice. Then, the slides were immersed in a freshly prepared lysing solution for a minimum of 2 h. After that, slides were submerged in fresh and chilled electrophoresis buffer (pH > 13, 1 mM EDTA, and 0.3 M NaOH) for 20 min prior to electrophoresis to allow the DNA to unwind. The electrophoresis was carried out at 0.3 A and 25 V for 20 min followed by the neutralizing of the slides in Tris buffer (pH 7.5, 400 mM). The slides were stained with ethidium bromide for fluorescent observation. CASP LAB was used as an image analyzer software, to examine comets. Randomly, 50 comets were scored per slides.

### 2.11 Statistical analysis

GraphPad Prism software version 7.0 was used for statistical analysis. All the findings are expressed as mean ± standard deviation. Two-way ANOVA, followed by Tukey's multiple comparisons test, was used to detect the significant difference in means within and between treatments. The results with *p*-value < 0.05 (*p* < 0.05) were considered statistically significant. R-software was used for Pearson's correlation for data analysis.

## 3 Results

**Figure 1:**
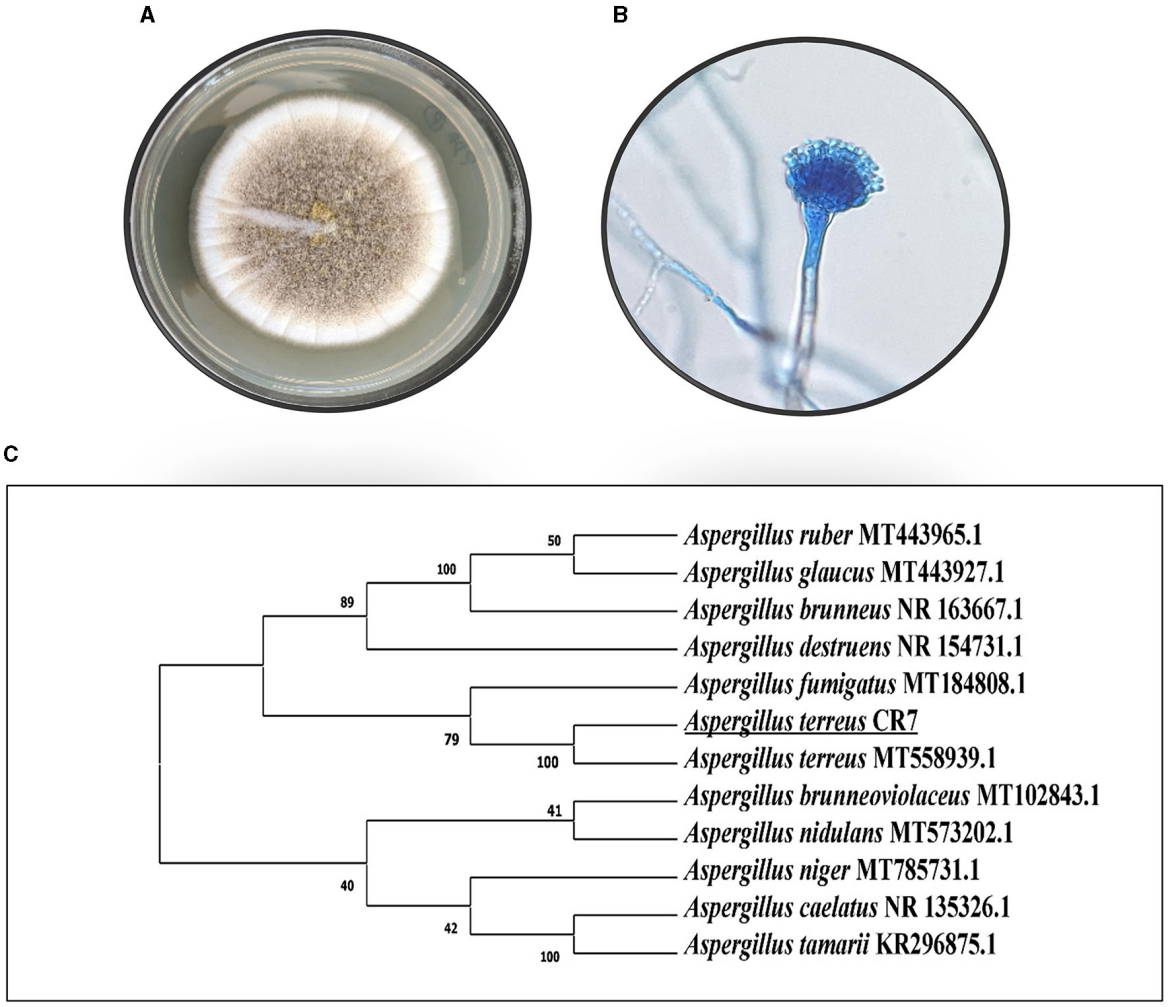
Morphology of CR7 **(A)** colony on PDA, **(B)** conidia and conidiophore, **(C)** phylogenetic tree presenting the position of CR7 on the basis of ITS1-5.8S rDNA-ITS2 gene sequence analysis.

In the present study, 15 endophytic cultures were isolated from different parts of *C. roseus* and screened for salt tolerance and plant growth promotion abilities, viz. IAA production, phosphate solubilization, siderophore, HCN, and ammonium production. An isolate CR7 exhibiting high salt tolerance (up to 15% NaCl) and plant growth promotion activities was selected for further studies. The culture produced high level of IAA and was also positive for phosphate solubilization, HCN, and ammonium production. No siderophore production was detected. Based on preliminary screening, endophytic isolate CR7 was selected and identified according to standard taxonomic key including colony diameter, color, and morphology of hyphae and conidia. The colonies were fast growing reaching a diameter of 4 cm after 5 days on PDA plates when incubated at 30°C, sporulating, cinnamon brown in color, darkening upon age and showed reverse pigmentation (Figure 1A). The conidial heads were compact, biseriate, and columnar. Conidia were small, round, smooth walled, and light brown in color (Figure 1B). Characterization of culture CR7 at molecular level was carried out by amplification of internal transcribed spacer (ITS) regions including 5.8S rDNA and DNA sequencing. After sequencing, a sequence of 484 bp was obtained which has been deposited in GenBank under accession number OQ353072. The sequence on alignment with homologous nucleotide sequences obtained from type strains revealed maximum similarity (100%) with *A. terreus* (Figure 1C). The culture has been submitted to National Center for Microbial Resource (NCMR) at National Center for Cell Science (NCCS), Pune, Maharashtra, India, under accession number NFCCI5502.

### 3.1 Estimation of IAA production

Selected isolate, *A. terreus* CR7, produced IAA in culture medium supplemented with tryptophan which was indicated by change in color of the supernatant to pink-red after the addition of Salkowski reagent. The effect of different concentrations of tryptophan and incubation time on the production of IAA was studied. Maximum IAA production of 23.43 ± 0.79 μg/ml was observed on 4th day of incubation at 10 mg/ml tryptophan (Supplementary Figure S1). Different concentrations of tryptophan [*F*_(4, 70)_ = 875.53; *p* < 0.05] and incubation period [*F*_(6, 70)_ = 641.87; *p* < 0.05) significantly affected IAA production.

### 3.2 Phosphate solubilization

The formation of clear zone around the colony in Pikovskaya's agar plates indicated the ability of the culture CR7 to solubilize phosphate. Phosphate solubilization index (PSI) was determined to be 1.044 ± 0.01. Soluble phosphate was determined by inoculating the culture in Pikovskaya's broth. The concentration of soluble phosphate in the culture filtrate varied with incubation time. The amount of available phosphate in the medium reached a peak (133.63 ± 6.40 μg/ml) after 21 days of incubation, thereafter decreasing to 31.99 ± 1.60 μg/ml on 35th day (Supplementary Figure S2A). The pH also varied, corresponding to the concentration of soluble phosphate (Supplementary Figure S2B). The pH gradually decreased to 4.06 ± 0.01 on 21st day, which coincided with maximum soluble phosphate, rising thereafter on further incubation.

### 3.3 Salt tolerance of *A. terreus* CR7

The salt tolerance of *A. terreus* CR7 was determined by growing the culture on PDA medium supplemented with different concentration of NaCl (ranging from 5 to 15% at interval of 5%). The culture showed good growth at 15% NaCl, attaining a diameter of 3 cm after 10 days of incubation. The presence of salt also affected the colony morphology as pale yellow to buff colonies were observed in the presence of salt as compared to dark brown colonies in control medium (Supplementary Figure S3).

### 3.4 Estimation of 1-aminocyclopropane-1- carboxylate deaminase

The culture, *A. terreus* CR7, also showed the potential to grow on Dwarkin and Foster medium supplemented with ACC as a nitrogen source indicating its ability to produce ACC deaminase. Assay of ACC deaminase activity revealed 86.36 ± 2.70 μmol α-ketobutyrate/h/mg protein on 2nd day of incubation. FTIR spectra analysis further verified the conversion of ACC into α- ketobutyrate which showed peaks at 1,639 and 3,339 cm^−1^ confirming the presence of ketonic and amino functional groups, respectively, which are recognized as α- ketobutyrate (Sarkar et al., 2018) (Supplementary Figure S4).

### 3.5 Colonization of *A. terreus* CR7 in *V. radiata* and effect of salt stress on seedling growth

Effect of colonization of *A. terreus* CR7 on various morphological and biochemical parameters in *V. radiata* was assessed under both normal and salt stress conditions. Colonization percentage was observed to be 73.6%, 67.2%, and 44.6%, in root, shoot, and leaf, respectively. Endophyte-treated plants showed good growth under both normal and stress conditions compared with un-inoculated plants. Under salt stress, at all the applied concentrations of NaCl (150, 200, 250 mM), un-inoculated plants showed stunted growth compared with inoculated plants. At highest concentration of NaCl, 33% of the un-inoculated plants died, whereas in case of inoculated plants, 100% survival was observed.

#### 3.5.1 Effect of *A. terreus* CR7 on morphological parameters of *V. radiata* under salt stress

**Figure 2:**
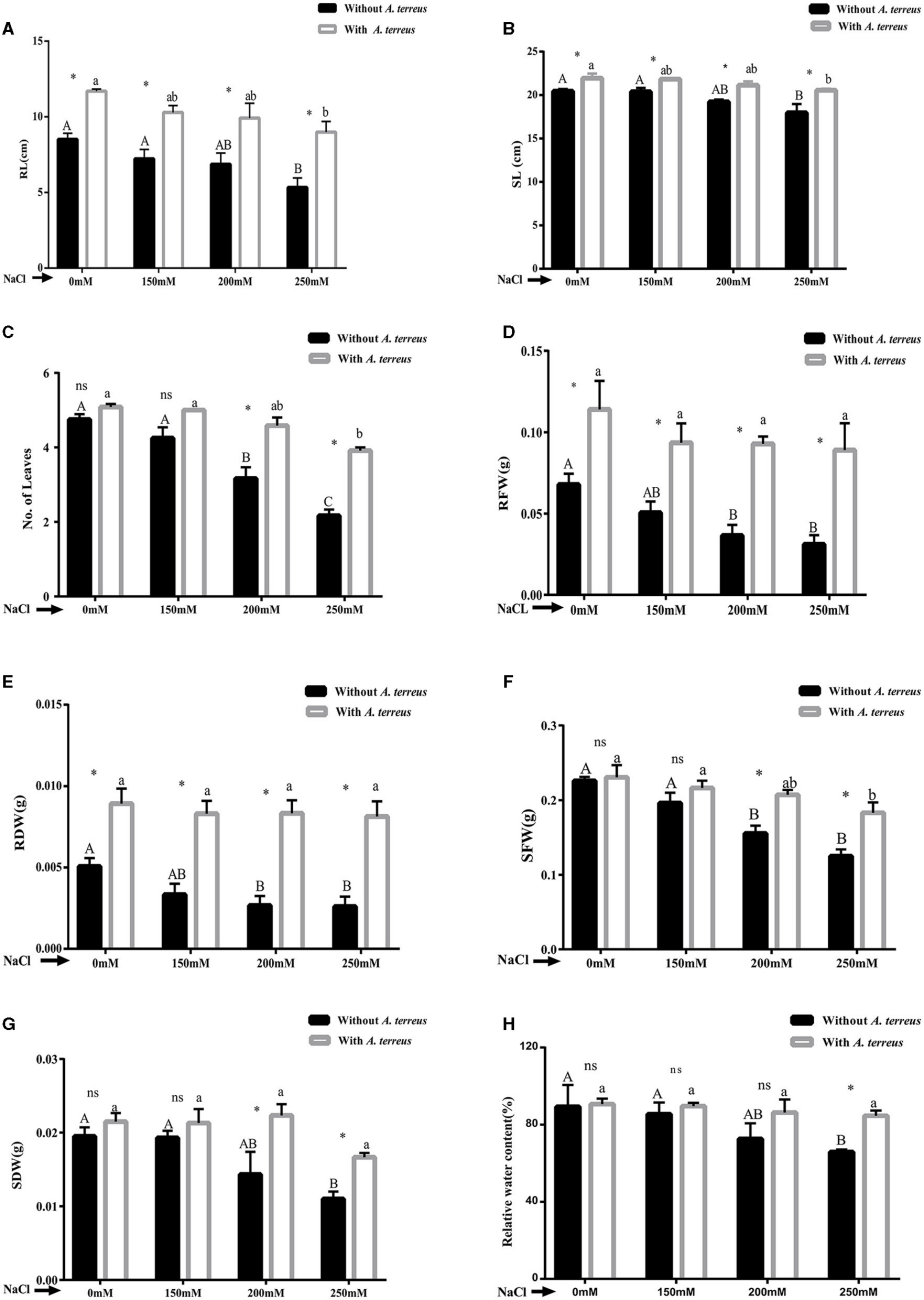
Effect of *Aspergillus terreus* CR7 on growth parameters: **(A)** root length (RL), **(B)** shoot length (SL), **(C)** numbers of leaves, **(D)** root fresh weight (RFW), **(E)** root dry weight (RDW), **(F)** shoot fresh weight (SFW), **(G)** shoot dry weight (SDW), and **(H)** relative water content of *Vigna radiata* plants under different salt stress. Data are expressed as mean ± SD. Same letters on the graph denote non-significant difference within a treatment. Capital letters for salt stress without *A. terreus* and small letters for salt stress with *A. terreus*. (*) denotes significant difference, and (ns) stands for non-significant among the treatments (with or without *A. terreus*).

*Vigna radiata* plants inoculated with *A. terreus* CR7 showed a significant (*p* < 0.05) improvement in morphological parameters under both normal and stress conditions. Under salt stress, at all the applied concentrations, root, shoot length, biomass, and number of leaves declined in un-inoculated plants whereas inoculation with *A. terreus* CR7 restored the growth attributes. At highest salt stress, the *A. terreus* CR7 inoculated plants showed significant (*p* < 0.05) increase of 68.35%, 14.47%, and 80.65% in root length, shoot length, and number of leaves, respectively, compared to un-inoculated plants (Figures 2A–C). Similarly, plants treated with *A. terreus* CR7 showed significant (*p* < 0.05) enhancement of 2.78-fold, 2.67-fold, 1.46-fold, and 1.55-fold in root fresh and dry weight, shoot fresh and dry weight, respectively, as shown in Figures 2D–G. The relative water content (RWC) of plants was significantly impacted by salt stress. At a salt stress of 250 mM, plants colonized with *A. terreus* CR7 showed significantly (*p* < 0.05) increased RWC (28.46%) compared with un-inoculated plants (Figure 2H).

#### 3.5.2 Effect of *A. terreus* CR7 on photosynthetic pigments of *V. radiata* under salt stress

**Figure 3:**
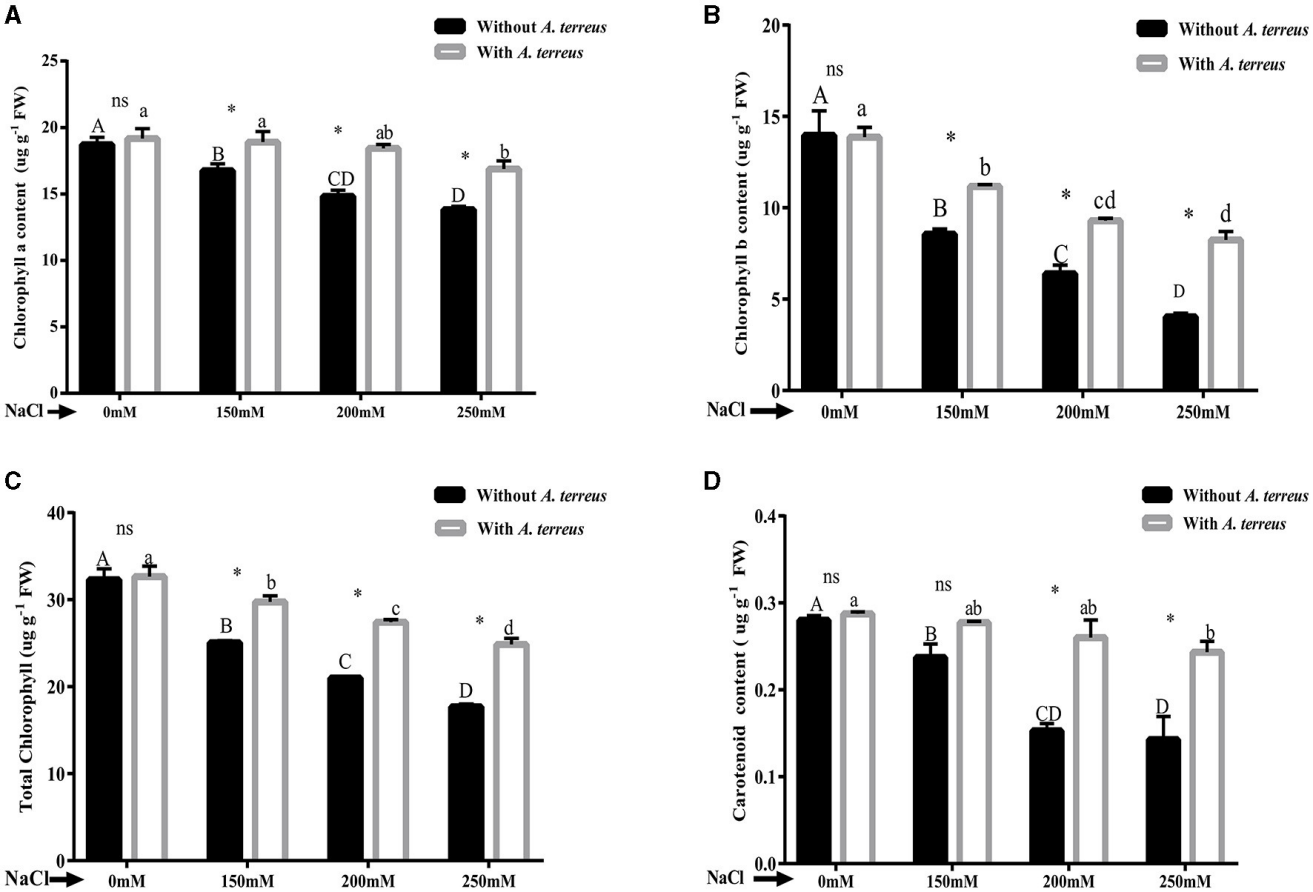
Effect of *Aspergillus terreus* CR7 on chlorophyll pigments attributes of *Vigna radiata* plants under different salt stress. **(A)** Chlorophyll “a,” **(B)** Chlorophyll “b,” **(C)** total chlorophyll, and **(D)** carotenoids. Data are expressed as mean ± SD. Same letters on the graph denote non-significant difference within a treatment. Capital letters for salt stress without *A. terreus* and small letters for salt with *A. terreus*. (*) denotes significant difference, and (ns) stands for non-significant among the treatments (with or without *A. terreus*).

Salt stress has been proven to adversely affect photosynthetic pigments. Chlorophyll a/b, total chlorophyll, and carotenoid were significantly (*p* < 0.05) improved in *A. terreus* inoculated plants as compared to un-inoculated plants at all applied salt concentrations. At highest salt stress of 250 mM NaCl, *A. terreus* inoculated plants showed significant increase of 1.22-fold, 2.04-fold, 1.40-fold, and 1.71-fold in chlorophyll “a,” chlorophyll “b,” total chlorophyll, and carotenoids compared to un-inoculated plants as shown in Figure 3. The restoration of photosynthetic pigments indicates the positive effect of *A. terreus* under NaCl stress.

#### 3.5.3 Effect of *A. terreus* CR7 on endogenous IAA, total protein content, and proline content under salt stress

**Figure 4:**
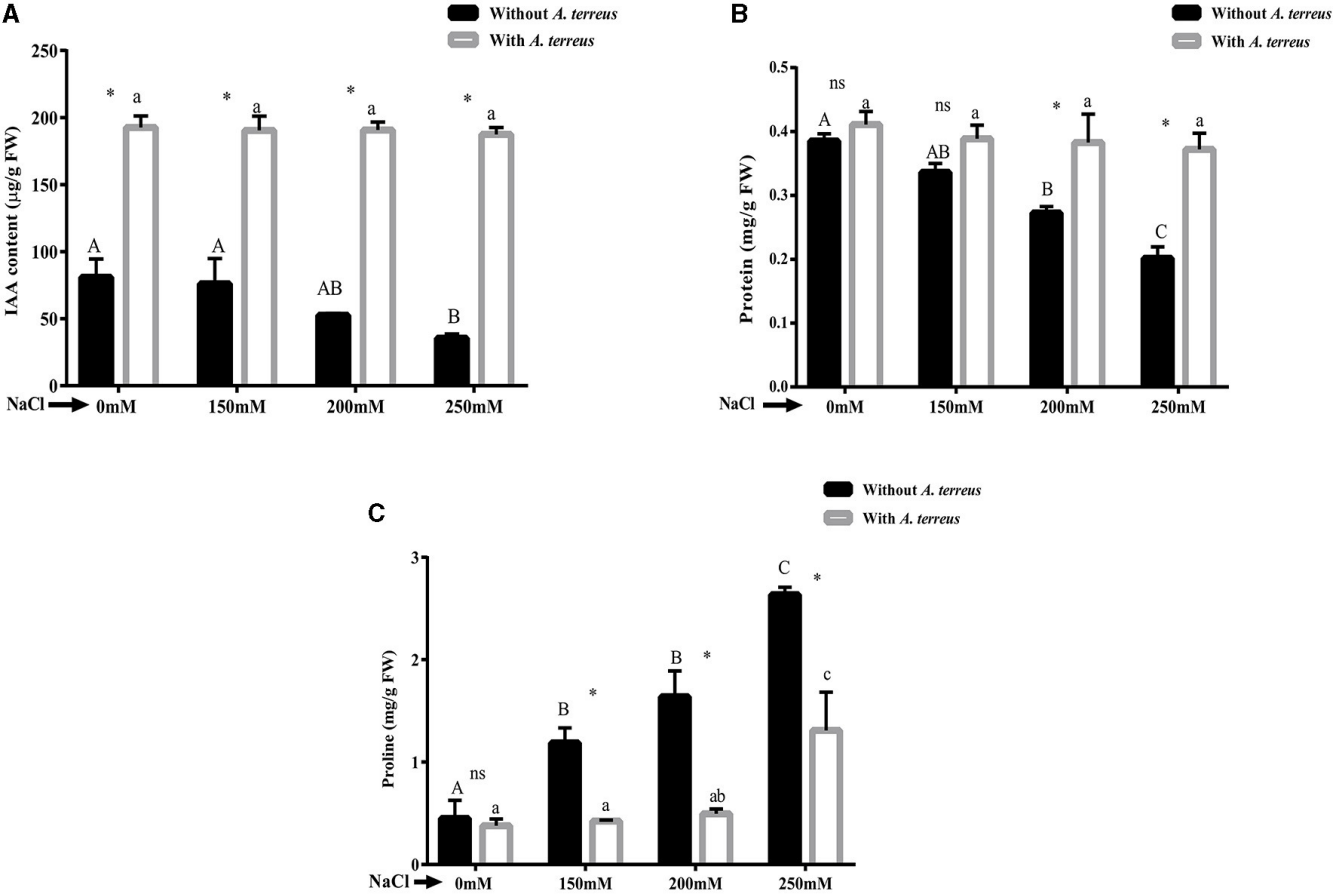
Effect of *Aspergillus terreus* CR7 on **(A)** endogenous IAA, **(B)** total protein content, and **(C)** proline of *Vigna radiata* plants under different salt stress. Data are expressed as mean ± SD. Same letters on the graph denote non-significant difference within a treatment. Capital letters for salt stress without *A. terreus* and small letters for salt stress with *A. terreus*. (*) denotes significant difference, and (ns) stands for non-significant among the treatments (with or without *A. terreus*).

The results exhibited significant (*p* < 0.05) decrease in endogenous IAA level in un-inoculated plants as compared to inoculated plants. At highest salt stress (250 mM NaCl), endogenous IAA increased 5.28-fold in inoculated plants compared to un-inoculated plants (Figure 4A). *V. radiata* inoculated plants also showed 85.07% enhancement in total protein content compared to un-inoculated plants (Figure 4B).

Proline is an osmo-protectant that accumulates in plants due to salinity stress. Proline content significantly (*p* < 0.05) increased in un-inoculated plants under salt stress. Treatment with fungus resulted in 50.19% decrease in proline content at 250 mM NaCl stress (Figure 4C).

#### 3.5.4 Effect of *A. terreus* CR7 on DPPH radical scavenging activity and phenolic content under salt stress

**Figure 5:**
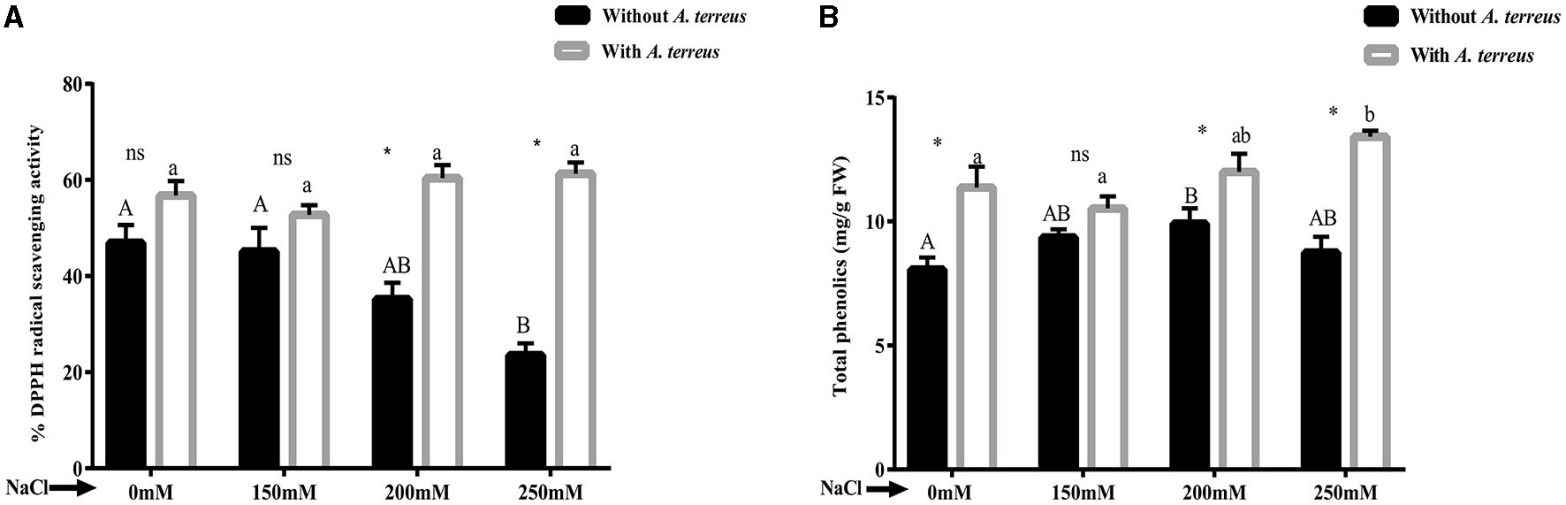
Effect of *Aspergillus terreus* CR7 on **(A)** DPPH radical scavenging activity and **(B)** total phenolic content in *Vigna radiata* plants under different salt stress. Data are expressed as mean ± SD. Same letters on the graph denote non-significant difference within a treatment. Capital letters for salt stress without *A. terreus* and small letters for salt stress with *A. terreus*. (*) denotes significant difference, and (ns) stands for non-significant among the treatments (with or without *A. terreus*).

The accumulation of antioxidants within plants can enhance resistance to the negative impacts of salt stress. Our study exhibited that DPPH radical scavenging activity was increased in *A. terreus* CR7 inoculated plants at all applied concentrations of salt compared with un-inoculated plants. At high salt stress (250 mM), DPPH radical scavenging activity increased 2.61-fold compared to the un-inoculated plants (Figure 5A). Moreover, phenolic content was also increased significantly (*p* < 0.05) in *A. terreus* CR7 inoculated plants. At the highest applied salt stress, total phenolic content increased by 53.46% in inoculated plants compared to un-inoculated plants (Figure 5B).

#### 3.5.5 Effect of *A. terreus* CR7 on oxidative stress parameters

**Figure 6:**
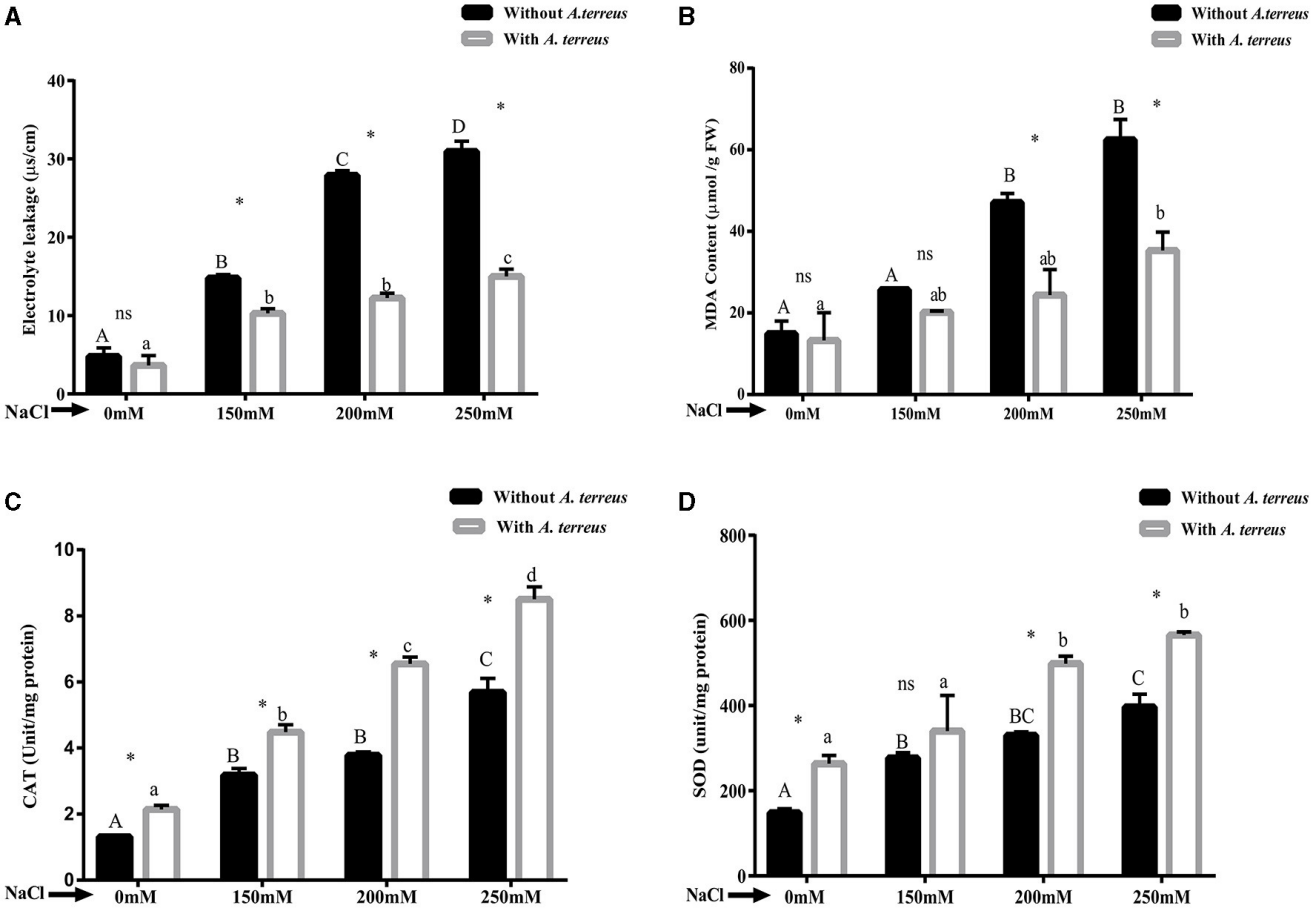
Effect of *Aspergillus terreus* CR7 on **(A)** electrolyte leakage, **(B)** MDA, **(C)** CAT, and **(D)** SOD in *Vigna radiata* plants under different salt stress. Data are expressed as mean ± SD. Same letters on the graph denote non-significant difference within a treatment. Capital letters for salt stress without *A. terreus* and small letters for salt stress with *A. terreus*. (*) denotes significant difference, and (ns) stands for non-significant among the treatments (with or without *A. terreus*).

The results demonstrated that application of salt stress resulted in reduction in oxidative stress parameters such as electrolyte leakage and MDA compared with un-inoculated plants. Application of *A. terreus* CR7 resulted in 51.50% reduction in electrolyte leakage at salt concentration of 250 mM NaCl (Figure 6A). Moreover, *A. terreus* CR7 inoculated plants also showed reduction of 43.45% in MDA content as compared to un-inoculated plants (Figure 6B). The results indicated a significant enhancement in CAT and SOD enzyme activity in inoculated plants compared with un-inoculated plants at all applied concentrations of NaCl. At highest concentration, an increase of 49.48% and 42.52% in CAT and SOD, respectively, was obtained in inoculated plants compared with un-inoculated plants (Figures 6C, D).

### 3.6 Determination of cell viability and oxidative stress using fluorescent microscopy

**Figure 7:**
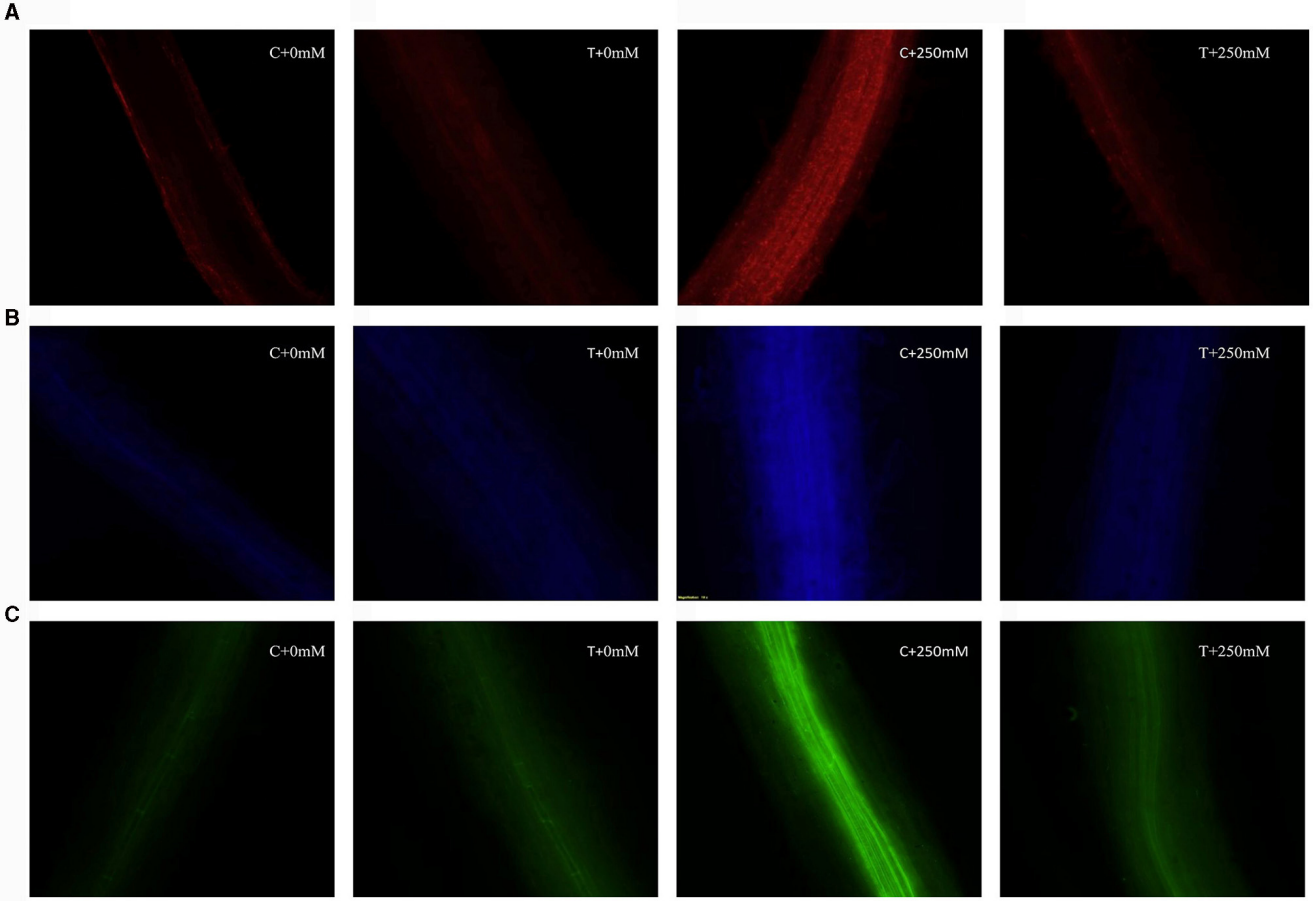
Fluorescent microscopic images, cell viability, **(A)** glutathione content, and **(B)** hydrogen peroxide localization **(C)**, without *Aspergillus terreus* 0 mM NaCl (C + 0 mM), with *A. terreus* 0 mM NaCl (T + 0 mM), without *A. terreus* 250 mM NaCl (C + 250 mM), and with *A. terreus* 250 mM NaCl (T + 250 mM).

Cell viability was determined on the basis of intensity of red color when stained with propidium iodide (PI). PI is a staining dye that is able to penetrate through disrupted cell membranes and stain nucleic acids, which can be visualized inside the dead cells as red fluorescent dots. Higher intensity of red color was observed in roots of plants exposed to salt stress. Treated plants exhibited less fluorescence intensity indicating higher cell viability and protective effect of *A. terreus* CR7 (Figure 7A). The roots of seedlings of *V. radiata* were also dipped in MCB dye to tag glutathione. Glutathione content was identified as the intensity of blue color. Roots of plants subjected to extreme salt stress (250 mM) had a high intensity of blue color, indicating high levels of glutathione. Plants inoculated with *A. terreus* CR7 displayed lower blue color intensity under high salt stress, indicating a decrease in glutathione levels (Figure 7B). High salt stress also causes oxidative stress, which was measured by hydrogen peroxide localization employing dichlorodihydrofluorescein-diacetate (H_2_DCFDA) dye. The green fluorescence was enhanced in 250 mM NaCl-treated plants in contrast to control plants, indicating enhancement in H_2_O_2_ levels. However, the fluorescence was less in case of fungal colonized plants (Figure 7C).

### 3.7 Geno protective effect of *A. terreus* CR7

**Figure 8:**
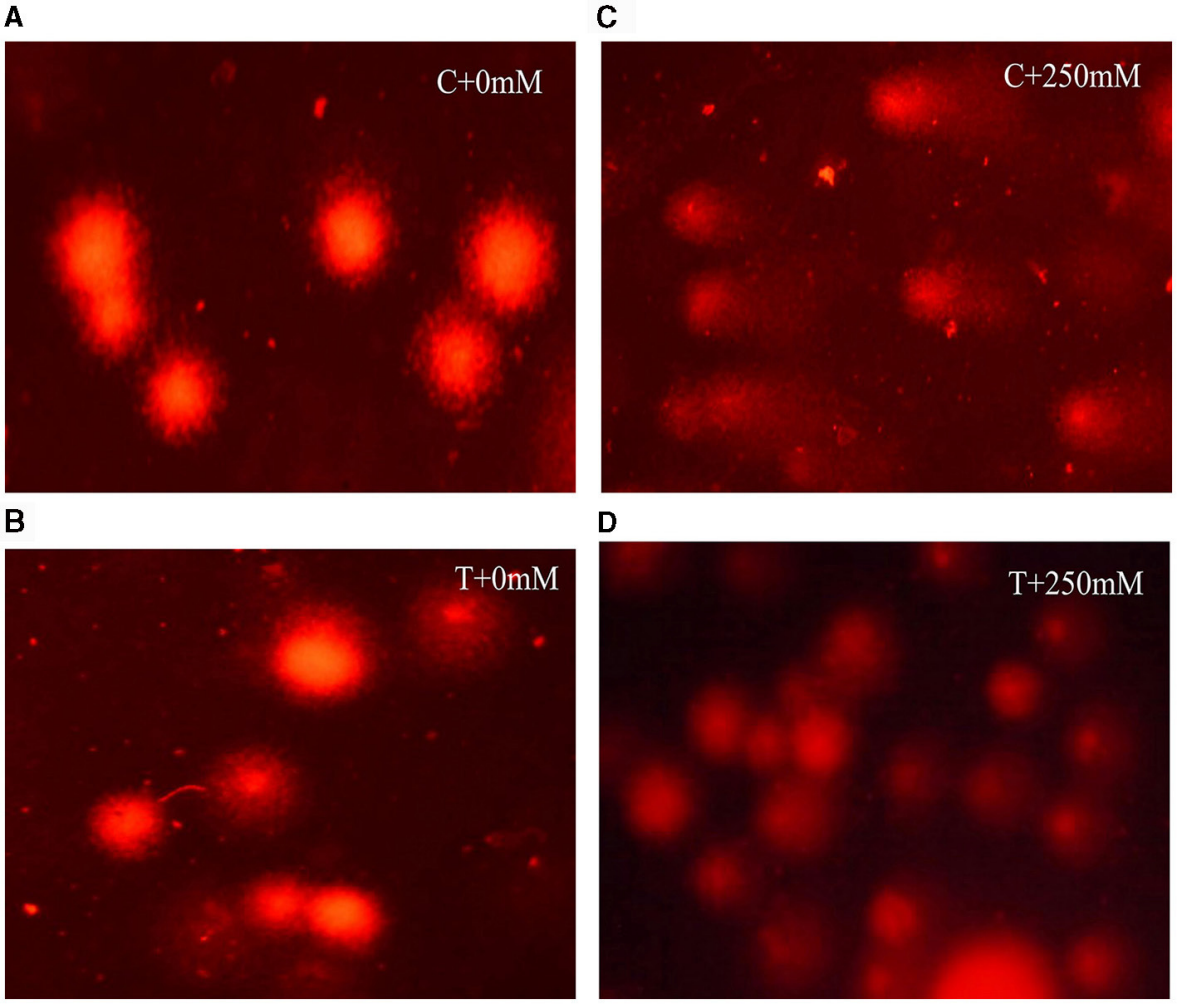
Fluorescent microscopic images showing different degree of DNA damage in roots of *V. radiata*, **(A)** without *Aspergillus terreus* + 0 mM NaCl, **(B)** with *A. terreus* + 0 mM NaCl, **(C)** without *A. terreus* + 250 mM NaCl, and **(D)** with *A. terreus* + 250 mM NaCl.

Comet assay was employed to determine the DNA damage in roots of un-inoculated and inoculated plants both under normal and stress conditions. Salt stress adversely affects the DNA in plants tissues. In this study, DNA damage was determined using comet assay parameters, viz. tail length, tail moment, and olive tail moment (Figure 8). At high salt stress (250 mM NaCl), DNA damage was evident in un-inoculated plants compared with inoculated plants (Figure 8C). In treated plants, significant (*p* < 0.05) reduction in tail length, tail moment, and olive tail moment, with a percent decrease of 19.87%, 19.76%, and 24.81%, respectively, compared with un-inoculated plants was observed (Table 1). The restoration of DNA damage in inoculated plants showed positive effect of *A. terreus* under salt stress.

**Table 1:** Effect of *Aspergillus terreus* CR7 on nuclear DNA in roots of *V. radiata* exposed to 0 mM and 250 mM NaCl stress.

	**Tail length (in** ****μ**m)**		**Tail moment (in** ****μ**m)**		**Olive tail moment**	
	**0 mM**	**250 mM**	**0 mM**	**250 mM**	**0 mM**	**250 mM**
Without *A. terreus*	16.79 ± 1.86a	39.35 ± 1.17a	14.38 ± 1.56a	35.61 ± 0.17a	7.23 ± 0.53a	20.81 ± 1.76a
With *A. terreus*	17.93 ± 0.46a	31.53 ± 0.52b	15.40 ± 0.96a	28.57 ± 0.53b	8.97 ± 0.41a	15.64 ± 0.35b

Data are expressed as mean ± SD. The values with distinct letters are significantly different (*p* < 0.05) according to Tukey's test.

### 3.8 Pearson's correlation analysis

**Figure 9:**
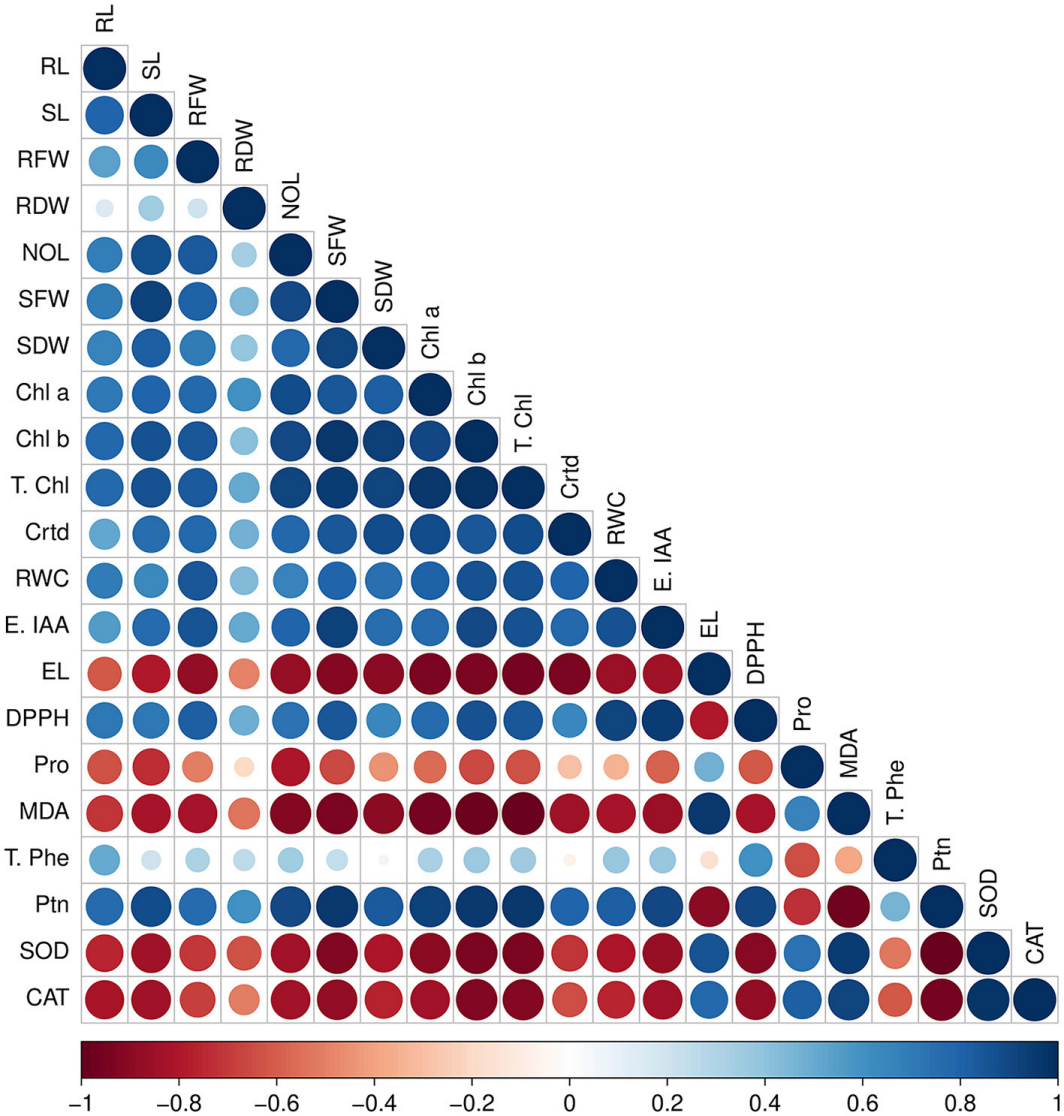
Pearson's correlation between antioxidants and biochemical traits with plant biomass parameters under various salt stresses; RL, root length; SL, shoot length; RFW, root fresh weight; RDW, root dry weight; NOL, number of leaves; Chl a, chlorophyll a; Chl b, chlorophyll b; T. chl, total chlorophyll; Crtd, carotenoids; DPPH, radical scavenging capacity; SOD, superoxide dismutase; CAT, catalase; Endo IAA, endogenous IAA; T. Phe, total phenolic content; Ptn, total protein; RWC, relative water content; EL, electrolyte leakage; MDA, malondialdehyde; Pro, proline. Red color showed negative correlation, and blue color showed a positive correlation.

Pearson's correlation analysis was done among the antioxidants and biochemical parameters with plant biomass under salinity stress (Figure 9). In *V. radiata* plants,photosynthetic pigments (chlorophyll a, chlorophyll b, total chlorophyll, and carotenoids) were found to have substantial positive correlation with root and shoot length, number of leaves, fresh and dry weight of root and shoot. The plant biomass enhanced with increase in these parameters. Similarly, biochemical attributes, viz. relative water content, endogenous IAA, DPPH radical scavenging capacity, and protein content, showed a positive correlation with plant biomass. In contrast, the antioxidant enzymes catalase, superoxide dismutase, MDA, electrical conductivity, and proline were found to be in a highly negative correlation with plant biomass.

## 4 Discussion

One of the greatest challenges facing agriculture worldwide and affecting food security is salinity of the soil. Plant–microbe interactions are emerging as a vital strategy for alleviating salt stress and restoring crop sustainability. Plant growth-promoting endophytic microbes have the ability to mitigate the effect of salt stress and enhance plant growth and nutrient uptake (Kumar et al., 2020; Gupta et al., 2021; Choudhary et al., 2022). A survey of literature revealed that many endophytic fungi have been reported to possess plant growth promotion potential (Numponsak et al., 2018; Khushdil et al., 2019; Mehmood et al., 2019; Javed et al., 2020). In the present study, endophytic fungi were isolated and evaluated for plant growth promotion ability and salt tolerance. On the basis of preliminary screening, a culture CR7 was selected which showed good plant growth promotion activities and exhibited halotolerance. The culture CR7 was identified as *A. terreus* on morphological and molecular basis. In the present study, selected isolate *A. terreus* CR7 produced high amount of IAA, phosphate solubilization potential, and also exhibited HCN and ammonium production. IAA production and phosphate solubilization ability of *A. terreus* have been also reported by Khushdil et al. (2019) and Khan et al. (2022). In our study, it was found that isolate *A. terreus* CR7 was able to grow in high salt concentration up to 15% NaCl. Isolates of *A. terreus* with ability to grow in up to 0.87 and 2.9% NaCl have been reported by Khushdil et al. (2019) and Khan et al. (2022), respectively. In the present study, no siderophore production was observed in the isolated culture, but other workers have documented the production of siderophore by *A. terreus* (Khan et al., 2022). ACC is precursor of ethylene, which has a significant negative impact on growth and development of plants (Saberi Riseh et al., 2021; Gamalero and Glick, 2022; Shekhawat et al., 2022). ACC deaminase enzyme mitigates the effect of ethylene and metabolizes ACC to ammonia and α-ketobutyrate. In this study, *A. terreus* CR7 produced high amount of ACC deaminase (86.36 ± 2.70 μmol α -ketobutyrate/h/mg protein). This was confirmed by FTIR analysis which demonstrated that ACC was metabolized by ACC deaminase as peaks corresponding to α- ketobutyrate and ammonia were observed. Numerous studies have reported that endophytic fungi produce ACC deaminase enzyme and mitigate the negative impact of salt stress in plants (Afridi et al., 2019; Zhang S. et al., 2019; Ji et al., 2021; Khan et al., 2022). The effect of *A. terreus* CR7 in promoting growth and mitigating salt stress was evaluated further in *V. radiata* plants using pot experiment. *Aspergillus terreus* CR7-associated plants showed significant enhancement in all the growth parameters inculding root length, shoot length, number of leaves, and fresh and dry biomass. Improvement in growth attributes by *A. terreus* has also been reported by other workers in wheat, pearl millet, maize, and rice by Khushdil et al. (2019), Khan et al. (2022), and Siddiqui et al. (2022).

A biochemical marker for the behavior of plants under salt stress is decrease in levels of photosynthetic pigments *viz*. chlorophyll a, b, and total chlorophyll. Under salt stress, a significant amount of Na^+^ ions may accumulate in chloroplast, inhibiting photosystem II and preventing photosynthesis, which ultimately has an impact on plant growth (Jansen et al., 1996; Sudhir and Murthy, 2004). In salt stress, ethylene production also increases, which is responsible for blocking the pathway for biosynthesis of chlorophyll. Carotenoids also play a role in defense mechanisms, by scavenging singlet oxygen or shielding chlorophyll from the damaging effects of photooxidation. Under salt stress, decrease in carotenoid content causes an increase in ROS production, which retards growth of plant by inducing oxidative damage (Behera et al., 2002; Ashraf and Harris, 2013). In our study, it was observed that under salt stress, chlorophyll content was significantly increased in inoculated plants compared to un-inoculated plants. The results shown in this study are in accordance with previous findings, where inoculation with halotolerant endophytic fungi increased the content of chlorophyll in various plants, *viz*. rice, maize, soyabean under salinity stress etc. (Jogawat et al., 2013; Asaf et al., 2018; Jan et al., 2022). The possible mechanism reported for this protection is inhibition of uptake of Na^+^ ions by fungal inoculated plants, which raises the content of chlorophyll and carotenoid in *V. radiata* plants under salt stress (Asaf et al., 2018). Salt stress also reduces the ability of plant cells to store water, which shows effect on leaf water content, and results in a build-up of Na^+^ ions inside plant cells (Zhao et al., 2021). The lower relative water content exhibits negative osmotic imbalance ultimately inhibiting plant growth (Hardoim et al., 2008). In this study, *A. terreus* CR7 inoculated plants had higher relative water content compared with un-inoculated plants. Similar results have also been reported by Gul Jan et al. (2019) that showed increase in relative water content in endophytic fungi inoculated plants under salt stress. Salt stress also modulates the levels of endogenous IAA. The results showed that the amount of endogenous IAA in un-inoculated plants reduced under salt stress, while *A. terreus* CR7 inoculated plants showed good endogenous IAA production. Increase in production of endogenous IAA in *V. radiata* could also be due to augmentation with IAA produced by *A. terreus* CR7.

Endophytic fungi are able to alleviate the negative impacts of salt stress by boosting a variety of physiochemical and biochemical processes in plants, such as production of antioxidant enzymes and secondary metabolites (Verma et al., 2022). In this study, *A. terreus* CR7 inoculated plants had a substantially higher DPPH radical scavenging activity compared with un-inoculated plants. Moreover, production of secondary metabolites such as phenolic compounds is another essential strategy for avoiding stress-related changes. Phenolic compounds can remove ROS from a stressful environment and prevent membrane deterioration. An increased production of phenolics in fungal inoculated plants under salt stress has also been demonstrated by other researchers (Khushdil et al., 2019; Mehmood et al., 2019). The present study also demonstrated that *A. terreus* CR7 increased the content of phenol in inoculated plants under salt stress. The increase in antioxidant activity, as revealed in DPPH radical scavenging assay, could also be due to increased phenolic content. Another important osmoprotectant molecule shown to increase under stress is proline. Proline content was also found to be enhanced in salinity-stressed plants, which decreased after inoculation with *A. terreus* CR7. Similar results showing reduction in proline content after fungal inoculation have been reported by other workers (Abdelaziz et al., 2019; Gul Jan et al., 2019; Badawy et al., 2021). On the contrary, increase in proline content in fungal inoculated plants has also been reported (Jan et al., 2022; Khan et al., 2022). The observed decrease in the production of proline in the present study could be due to antioxidant activity of endophytic fungus, which mitigated the osmotic stress by scavenging ROS.

The generated oxidative stress under high salt conditions is also reflected in biochemical alterations that cause salinity-induced harm to biomolecules and plant cell structures (Ma et al., 2020). Increase in the oxidation of unsaturated fatty acids by ROS leads to various products such as malondialdehyde, which is an indicator of oxidative stress-induced membrane damage (Zhang et al., 2016). In the present study, under salt stress, the amount of MDA increased reflecting ROS production in salt-stressed plants. After colonization with *A. terreus* CR7, MDA level was decreased which could be due to scavenging of ROS by the endophytic fungus. Increase in MDA content in salt stress and its mitigation by endophytic fungi have also been reported by other workers (Ta¨ıbi et al., 2016; Hnilickova et al., 2021). Salt stress can also raise electrolyte discharge within plant tissue, which can further damage or displace the cellular membrane stability (Demidchik et al., 2014). In the present study, *A. terreus* CR7 inoculated plants showed lower electrolyte leakage compared with un-inoculated plants, indicating decreased levels of stress. Catalase and superoxide dismutase are antioxidant enzymes that can mitigate the detrimental effect of ROS in stressful conditions. Numerous studies have reported the activation of antioxidant enzyme system mediated by endophytic fungi under salt stress (Radhakrishnan et al., 2015; Li et al., 2017). In our study also, it was observed that the presence of *A. terreus* CR7 in *V. radiata* plants under salt stress increased the activity of antioxidant enzymes as compared to the non-inoculated plants.

The cell viability and oxidative stress in roots were qualitatively examined using *in vivo* histochemical labeling with fluorescent dyes. Propidium iodide (PI), a fluorescent dye, is used to stain DNA molecules and evaluate cell membrane damage. PI enters through damaged cell membranes and intercalates with DNA, resulting in red fluorescence. In the current study, high salt stress affected the viability of cells causing damage, which was visualized as higher intensity of red color. The intensity of red color was reduced in case of fungal colonized roots indicating protective effect. Similar results showing impact of salinity-induced stress on the cell membrane in pea root tips have been reported (Alharbi et al., 2022). Under salt stress, oxidative stress rises which leads to the accumulation of hydrogen peroxide in plants, which can be detected after staining the root tissues with fluorescent dye 2′, 7′-dichlorodihydrofluorescein diacetate (H_2_DCFDA) (Liu et al., 2020). H_2_DCFDA is most extensively used dye for detecting cellular H_2_O_2_. It is cell permeable, diffuses into cells, and is deacetylated by cellular esterases to generate 2′, 7′-dichlorodihydrofluorescein (H_2_DCF). In the presence of H_2_O_2_, H_2_DCF is quickly oxidized to 2′, 7′-dichlorofluorescein (DCF), resulting in green fluorescence (Galluzzi and Kroemer, 2014). In the present study, green fluorescence was enhanced in salt-stressed plants in contrast to control plants, indicating increase in H_2_O_2_ levels. The intensity of green color reduced in case of fungal colonized plant roots. Localization of glutathione (GSH) in plant roots was visualized using monochlorobimane (MCB) dye. MCB is a cell permeant dye with high affinity for GSH. Endogenous glutathione-S-transferase (GST) enzymes catalyze the dye's reaction with GSH, resulting in blue fluorescence (Gautam et al., 2021). In this study, amount of glutathione was increased under salt stress as indicated by increased intensity of the blue color in comparison with control. The fungal colonized root showed reduced blue color intensity indicating reduction in level of glutathione.

Salt stress has been shown to increase formulation and accumulation of ROS that results in oxidative stress and cause DNA damage (Nisa et al., 2021). Salinity-induced ROS-dependent DNA damage and alteration in plants has been reported by Alotaibi (2021) and Darwish et al. (2023). Comet assay was used to examine the damage to DNA caused by salt stress and the protective effects of *A. terreus* CR7. This assay, commonly referred to as a single-cell gel electrophoresis assay, is an efficient way to identify DNA damage in individual cells. The principle behind this is that under the influence of an electric field DNA fragments, which are negatively charged move toward anode resulting in comet-like appearance. The degree of DNA strand breakage is estimated by the extent of migration. Genotoxic damage induced by salt stress can be assessed using various parameters *viz*., tail length, tail moment, and olive tail moment (Lu et al., 2017). Comet assay has also been employed by other workers to assess salinity-induced DNA damage (Alotaibi, 2021; Darwish et al., 2023). In this study, all the examined DNA damage parameters viz., tail length, tail moment, and olive tail moment were improved in *A. terreus* CR7 treated plants compared to control plants. This is the first study documenting the alleviation of salt stress in *V. radiata* by an endophytic *A. terreus* CR7. In addition to this, the genoprotective effects of endophytic fungi under salt stress are also being reported for the first time.

## Data availability statement

The datasets presented in this study can be found in online repositories. The names of the repository/repositories and accession number(s) can be found in the article/Supplementary material.

## Author contributions

PChau: Data curation, Formal analysis, Investigation, Methodology, Writing—original draft. MandS: Data curation, Formal analysis, Methodology, Writing—original draft. AS: Data curation, Formal analysis, Methodology, Writing—original draft. MangS: Formal analysis, Methodology, Writing—original draft. PChad: Supervision, Writing—review & editing. AK: Data curation, Investigation, Methodology, Supervision, Validation, Visualization, Writing—review & editing.
