# Supplementary material for: Halotolerant and plant growth-promoting endophytic fungus Aspergillus terreus CR7 alleviates salt stress and exhibits genoprotective effect in Vigna radiata
Source: Front Microbiol. 2024 Feb 9;15:1336533. doi: 10.3389/fmicb.2024.1336533 (PMC10884769; doi:10.3389/fmicb.2024.1336533)
Supplement: Supplementary file 1 [file Data_Sheet_1.PDF]

Supplementary Material

1 SUPPLEMENTARY FIGURES

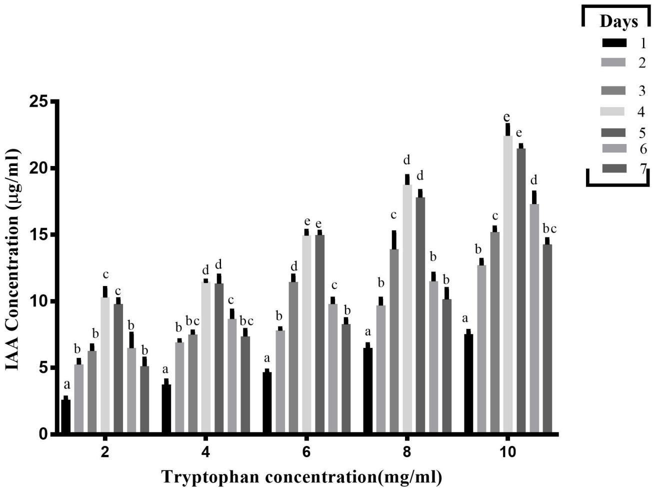

Figure S1. Effect of incubation time and different concentrations of tryptophan on IAA production. Data are expressed as mean ± SD. The values with distinct letters are significantly different ($p < 0.05$) according to Tukey's test

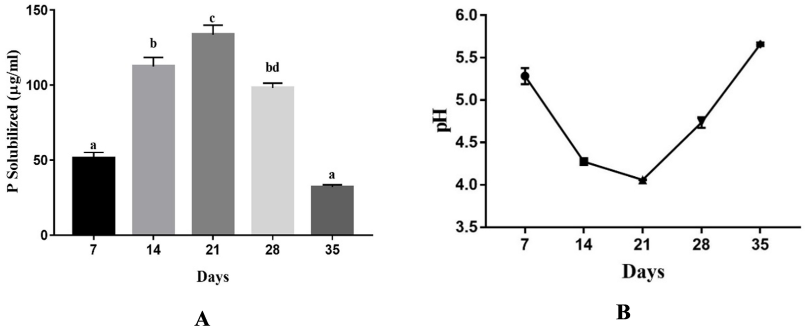

Figure S2. Phosphate solubilization by A. terreus CR7 (**A**) Effect of incubation time on phosphate solubilization. (**B**) Effect of incubation time on pH of culture filtrate. Data are expressed as mean ± SD. The values with distinct letters are significantly different ($p < 0.05$) according to Tukey's test

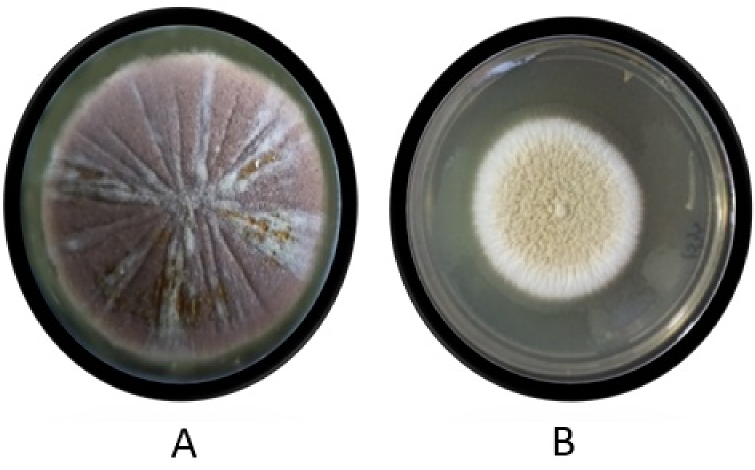

Figure S3. Growth of *A. terreus* CR7 on potato dextrose agar medium (**A**) without NaCl (**B**) with 15% NaCl

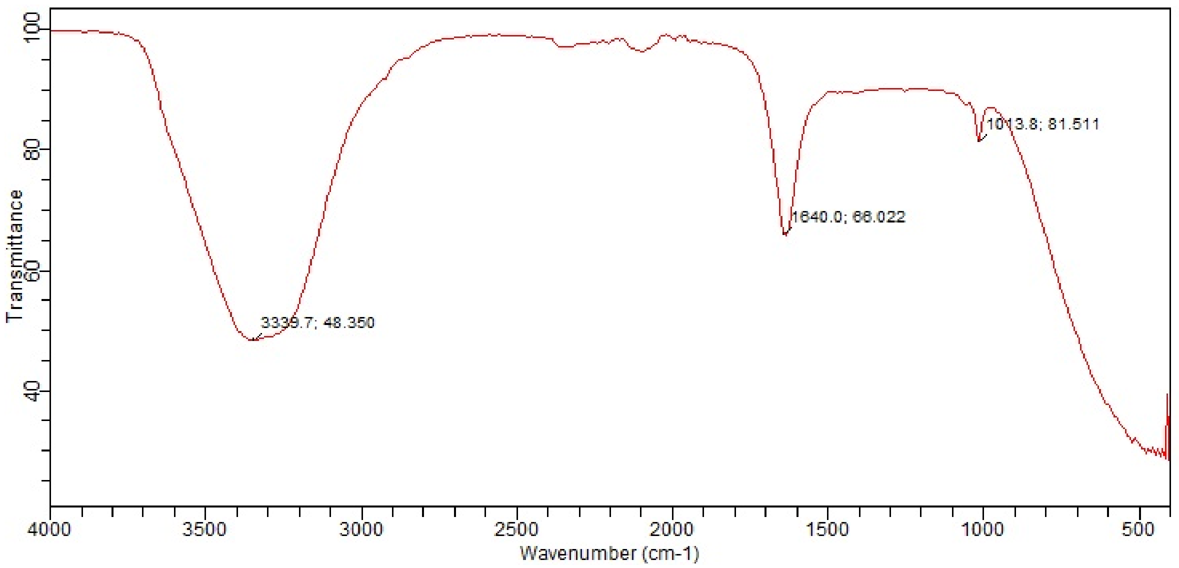

Figure S4. FTIR spectra of α-ketobutyrate in the strain *A.terreus* CR7
